# Quantitative Analysis of the *P65* Gene Expression in Patients with Coloroctal Cancer

**Published:** 2007-12

**Authors:** E. Balcerczak, M. Balcerczak, M. Mirowski

**Affiliations:** 1 *Department of Pharmaceutical Biochemistry, Laboratory of Molecular Biology and Pharmacogenomics, Lodz, Poland;*; 2 *Department of Surgery District Hospital, Leczyca, Poland*

**Keywords:** colon cancer, *P65* gene, real-time PCR

## Abstract

*P65* gene expression level was determined in colon cancer cases by means of real-time PCR. 51 cases of colorectal carcinomas showing positive RT-PCR signals for *P65* gene expression selected from 109 frozen samples were further investigated by quantitative real-time PCR. *P65* levels were higher in cancer with metastases to lymph nodes and distant metastases. Higher levels were observed in more advanced cases classified as III and IV according to pTNM classification. In two groups of patients with vessel invasion and absence of lymphocytes in tumour tissue, the presence of *P65* expression correlated with shorter survival time. Quantitative results confirmed that *P65* gene expression in colon cancer is engaged in the process of metastasis formation and could be correlated with worse prognosis for the patients.

## INTRODUCTION

A 65 kDa protein (*P65*) is expressed by many types of tumour cells. *P65* was originally isolated from the MCF-7 human breast cancer cell line (1). The presence of the 65 kDa protein was observed not only in tumour tissue but also in the sera of patients diagnosed with different types of neoplasm including colon cancers (2-6). Blood level of *P65* in patients suffering from cancer was significantly higher in comparison to healthy men. It was also showed that in colon cancer, estimation of *P65* level in serum parallel to CA19-9 and CEA improved the threshold of cancer detection when compared to CEA or to CA19-9 levels alone (7). Such results were obtained with the use of anti-*P65* monoclonal and polyclonal antibodies raised against this protein (5, 6, 8). Immunohistochemical analysis performed in cases of breast carcinomas revealed both cytoplasmic and nuclear localization of this protein in the tumour cells (9).

Qualitative analyses of *P65* gene by reverse-transcriptase PCR were performed in various types of leukemia (2), breast (10) and prostate cancers (3). In breast cancer *P65* expression was generally connected with small tumors without metastases in regional lymph nodes, while the absence of *P65* expression was observed in cases classified as fibroadenoma (10). Similarly, in prostate cancers *P65* was mainly present in well-differentiated tumors. These results suggested that expression of *P65* may correlate with a better prognosis for these patients (3, 10). In contrast, in a study of a population of 109 colon cancer cases the results were quite different. In this group *P65* gene expression correlated with grading, clinical staging and other histological features (4). Unexpectedly, significant statistical correlations between the presence of *P65* gene expression and the depth of tumor invasion (T3, T4), presence of lymph node metastases (N1-N2) and distant metastases (M1) with high clinical stages (C2, C3 and D according to Astler-Coller classification) and with vessel invasion were suggesting a poor prognosis (4). In this paper, gene expression was analyzed quantitatively by means of real-time PCR for the 51 colon cancer samples that had previously tested positive for *P65* expression.

## MATERIALS AND METHODS

### Human colon cancer tissues

Tissue specimens of colorectal carcinomas were obtained from the Oncological Centre of Lodz, Poland under the license of the local ethics committee (KE/286/05). 51 cases for quantity analysis were chosen from the whole population (n=109) on the basis of analyses carried out by quality multiplex reverse transcriptase PCR (4). In this group, 23 cases of carcinomas were from women (mean age 60.5 ± 5) and 28 cases were from men (mean age 62.5 ± 5).

### Quantification of the level of *P65* gene expression by real-time–PCR

For quantitative analysis, RNA was isolated using a Total RNA Prep Plus Minicolumn Kit (*A&A Biotechnology, Poland*) based on RNA isolation methodology developed previously (11). All RNA samples were treated with DNAse (Sigma) to remove genomic DNA. The RNA quantity was calculated after measurement of absorbance. Then the same quantity of RNA for each sample were transcribed into cDNA using Enhanced Avian HS RT-PCR Kit (*Sigma*). Real-time PCR was performed using iCycler (Bio-Rad) and SYBR Green Jump™ Start Tag ReadyMix™ (*Sigma*) according to manufacturer’s instructions.

The *P65* primer set 5’-GGTCCACGGCGGACCGGT-3’ (forward) and 5’-GACCCCGAGAACGTGGTGCGC-3’ (reverse) and conditions used in the assay were previously described (10). The following reagents were used for thermocycling: 25 μl Jump Start Tag Ready Mix, 0.5 μl reference dye, 1 μl of forward primer (final concentration 0.2 μM), 1 μl of reverse primer, 2 μl magnesium chloride (final concentration 25 mM), 5 μl template cDNA, and water (final volume 50 μl.) Cycling parameters were hot starting at 98°C for 5 min, followed by 2 min initial denaturation at 94°C, followed by 35 cycles consisting of denaturation at 94°C, 1 min annealing at 54°C and 3 min extension at 72°C, followed by 7 min final extension at 72°C. As a loading standard, the expression of *β-actin* gene was quantified for each sample using 5’-GTGGGGCGCCCCAGGCACCA-3’ (forward); 5’-CTCCTTAATGTCACGCACGATTTC-3’ (reverse) primer set (12). The experiments with *P65* and *β-actin* were done not as a multiplex, but in the separated tubes during the same PCR. Because of this the final results are not given as relative ratios. The level of *P65* was monitored by measuring the increased fluorescence of SYBR Green through the PCR cycles and estimated at the threshold cycle for each analysis. *P65* expression levels were calculated on the basis of a standard curve (units used for preparing curve ng/μl) that was obtained by plotting known quantities of genomic *P65* DNA. Then data were transformed into Microsoft Excel. Experiments for all samples were carried out in triplicate.

### Statistical analyses

Statistical analyses were performed using the U Mann-Whitney test. Time-to-event distribution for survival in the whole population of 109 patients was estimated using the Kaplan-Meier method. The F-Cox test was used to test for differences in time-to-event distribution.

## RESULTS

### *P65* gene expression levels are related to clinico-histological features

109 of colon cancer cases were analyzed by conventional reverse transcriptase PCR, 51 of which the presence of *P65* gene expression. All positive cases were taken for further quantitative analysis using real-time PCR.

*P65* gene expression levels were compared with several clinicopathological parameters such as depth of tumor invasion (T), lymph node metastases (N), and distant metastases (M) (TNM classification).

**Figure 1:**
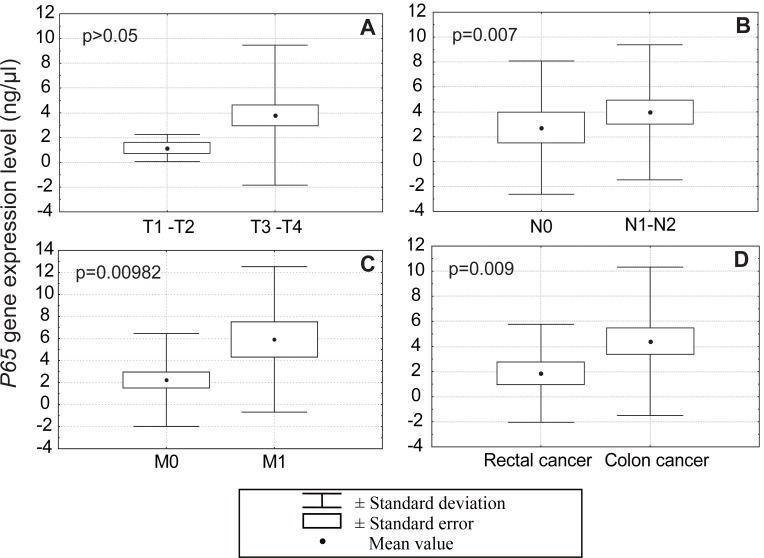
The levels of *P65* gene expression in T1, T2 and T3, T4 group (A), in cases without (N0) or with (N1 and N2) metastases to lymph nodes (B), in cases without (M0) or with (M1) distant metastases (C) and in rectal and colon cancers (D).

*P65* gene expression in the more advanced tumors (T3 and T4, deep wall penetration) was 4 ng/μl, while in the T1-T2 group lower levels of expression were recorded (1 ng/μl). No statistically significant correlation was observed between *P65* gene expression and tumor invasion depths (Figure 1A).

*P65* gene expression was analyzed in cases with and without lymph node metastases. In cases without lymph node invasion (N0) *P65* levels were 3 ng/μl, while in cases with lymph node metastases (N1-N2) *P65* mRNA levels were 4 ng/μl (Figure 1B). This difference was statistically significant (*p*=0.0395 U Mann-Whitney test).

In carcinomas with distant metastases the levels of *P65* expression were two times higher (M1: 6 ng/μl) than in the group of cancers without distant metastases (M0: 3 ng/μl) -Figure 1C. These differences were statistically significant (*p*=0.009872 U Mann-Whitney test).

*P65* levels were statistically lower in cancers originating in the rectum as compared to those originating in the colon (*p*=0.009 U Mann-Whitney test, Figure 1D).

Comparison of *P65* gene expression levels with pTNM staging showed that the more advanced cases (stages III and IV) had statistically significant higher levels of *P65* (4 ng/μl) than stages I and II (2 ng/μl) - *p*=0.0218 U Mann-Whitney test.

The cases without lymphocytes in tumor tissue showed higher levels of *P65* (4 ng/μl) then those with the presence of lymphocytes (2.5 ng/μl), but this difference was not statistically significant. Furthermore, in the group of tumors without vessel invasion the level of *P65* gene expression was lower (2 ng/μl) than in tumors with the presence of vassel invasion (4 ng/μl) but this difference was also not statistically significant. The data describing *P65* gene expression in relation to clinico-histological features are summarized in Table 1.

**Table 1:** Dependences between the numbers of *P*65 possitive cases in relation to clinico-histological features

Analyzed parameter		Number of cases with *P*65 gene expression	p

Histological type	Tubular adenocarcinoma	47	*p*=0.1 (Chi square test with Yates correction)
	Mucinous adenocarcinoma	4	
T	T1-T2	6	*p*=0.0003 (V square test)
	T3-T4	45	
N	N0	19	*p*=0.0001 (Chi square test)
	N1-N2	32	
M	M0	34	*p*=0.0012 (Chi square test with Yates, correction)
	M1	17	
Clinical stage, of disease	I-II	17	*p*<0.0001 (Chi square test)
	III-IV	34	
Vassel invasion	Absent	14	*p*=0.016 (Chi square test)
	Present	37	
Lymphocytes infiltration	Absent	33	*p*=0.084 (Chi square test)
	Present	18	

### *P65* expression and survival time

**Figure 2:**
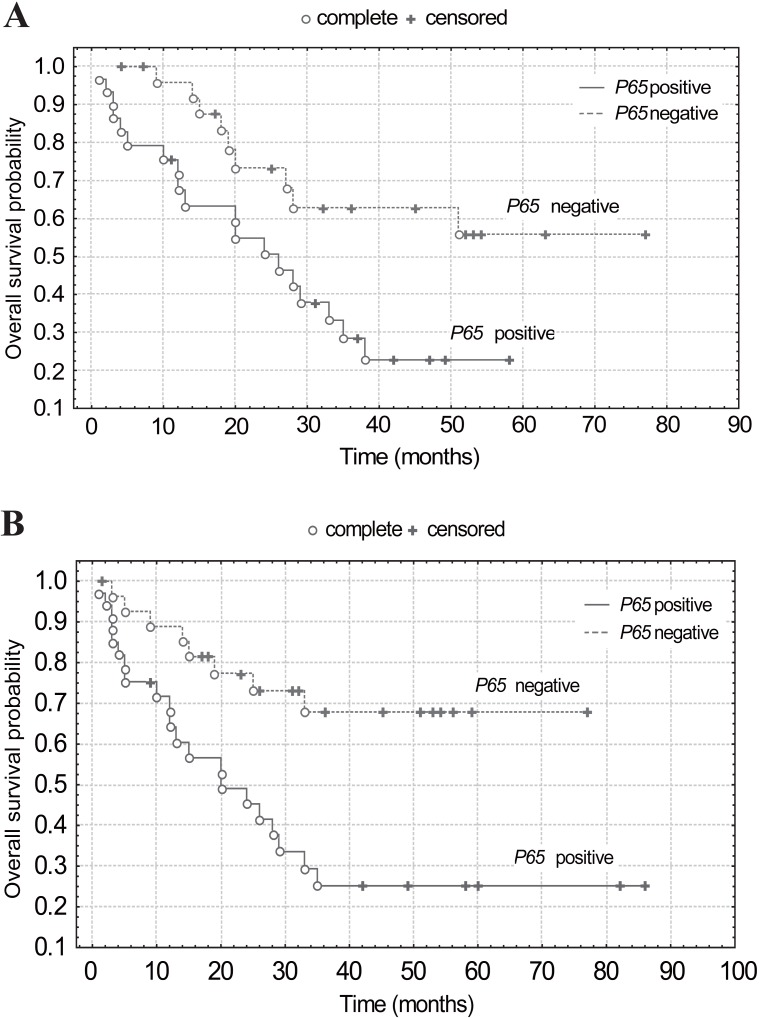
Differences in overall survival probability stratified by the *P65* gene expression in the group without limphocytes in tumor tissue (A), in the group with vessel invasion (B).

In the entire investigated population (n=109 patients), there was no statistically significant different in survival time comparing patients with and without *P65* gene expression in spite of the visible tendency for longer survival of those without *P65* gene expression (p>0.05). It is interesting to note that in the group of patients with vessel invasion (*p*=0.01396 F-Cox test, *p*=0.00267 log. rang test) and also in the group with the absence of lymphocytes in tumour tissue (*p*=0.02380 F-Cox test, *p*=0.01147 log. rang test) the presence of *P65* expression correlated with shorter survival time (Figure 2A and B). Further analysis of different variants didn’t show differences in survival time between *P65* positive and negative groups.

### *P65* gene expression level in cancer and adjacent, potentially healthy tissues

It may be of interest that 19 out of 51 investigated cases revealed *P65* expression in quality analysis in both neoplastic and adjacent, potentially healthy colorectal tissue. Quantitative studies showed that in adjacent, healthy tissue *P65* levels were statistically lower in comparison to cancer tissue (*p*=0.019).

## DISCUSSION

### The potential role of *P65* protein in cancer diagnosis and prognosis

Cancer of the large bowel is a serious and growing medical problem. Thus, there is a real need to identify factors engaged in development of colorectal cancer that could be useful in prognosis and therapy selection. The *P65* gene and its tumour-associated *P65* protein seem to be involved in neoplastic transformation and tumour progression in colon cancer (4, 7). The pathways involved are still unknown and the biological functions of *P65/P65* are not completely understood. On the basis of established partial amino acid and nucleotide sequences, homology to steroid receptors was revealed, especially among the DNA-binding domains (1, 13). However, other regions of the sequence do not show similarities. This suggests that *P65* may be either a new receptor with unknown ligand or a transcriptional factor. Interaction of *P65* with DNA can modulate transcription of some genes important for regulation of growth, development or cell differentiation (14, 15). This hypothesis is supported by the cytoplasmic and nuclear staining by anti-*P65* antibodies (9). Immunohistochemical analysis showed that *P65* cytoplasmic reactivity correlated with high levels of high estrogen and progesterone receptors level and with low clinical stage and grade. Nuclear manifestation of *P65* expression was connected with more advanced stages, particularly with node and distant metastasis and high grade (9).

### The potential role of *P65* gene expression in cancer diagnosis and prognosis

Qualitative studies using reverse transcriptase PCR showed that *P65* gene expression is not present in healthy tissues but can be found only in cancer tissues or in benign changes that can progress to malignant tumours (3, 10) These data indicated that expression of this gene is connected with neoplastic transformation, but not in all investigated cases, e.g. colon cancer where nearly half of carcinomas expressed *P65* (4). In these cases, the presence of *P65* was connected with metastases to lymph nodes and distant metastases, and these correlations were statistically important. Such results were confirmed in the current study by real-time PCR analysis, where the levels of *P65* were higher in more advanced cancers. It may prove *P65* may play important roles in metastatic progression of colorectal cancer that could be potentially useful in clinical practice. The most important is the correlation between high levels of *P65* and distant metastases. It should be noted that the cause of death in the colon cancer patients is often not the primary tumour but the secondary metastases to the liver. These changes are present in a clinically non-detectable form, known as micro-metastasis, in 50% of colorectal cancers cases at the time of diagnosis. For patients with high levels of *P65* without clinically manifested metastases more aggressive therapy may be indicated, and they should be monitored more closely.

### *P65* gene expression level as an early indicator of cancer formation

It should be noted that in 19 out of the 51 cases investigated *P65* was detected in both cancerous tissue and adjacent, healthy mucosa (classified by pathologist). Quantitative results showed significantly higher levels of *P65* in cancer tissue. Nearly 80% of these 19 cases were classified according to pTNM as III or IV. Such results may suggest that *P65* appeared earlier then typical neoplastic changes visible to the histopathologist and that the margin of surgical intervention should be increased. This is in agreement with previous, experimental studies where *P65* rapidly accumulated in the blood plasma of rats 14 days serum after partial hepatectomy and treatment with N-diethylnitrosamine (DEN) and reached a plateau by 3 weeks. These changes were earlier than any neoplastic minifoci in hepatoma visible by histopathologists (16).

Although it is too early to speculate about the molecular mechanisms of *P65*/*P65* action and its role in cancer formation and spreading, this study shows that high levels of *P65* gene expression correlate with advanced stage of disease in colon cancer. These results are in agreement with our earlier observation in breast cancer patients (9) where the presence of *P65* in cancer cell nuclei also correlated with formation of metastases. Certainly, *P65* protein and *P65* gene expression may behave in different ways in various types of tumors but their presence and elevated levels may have prognostic significance. However, further investigations are needed to establish the gene structure of *P65*, its genome localization, its role in tumor development and any diagnostic/predictive value of *P65/P65* in different cancers.
